# Efficient chiral synthesis by *Saccharomyces cerevisiae* spore encapsulation of *Candida parapsilosis* Glu228Ser/(*S*)-carbonyl reductase II and *Bacillus* sp. YX-1 glucose dehydrogenase in organic solvents

**DOI:** 10.1186/s12934-019-1137-6

**Published:** 2019-05-20

**Authors:** Jingxin Rao, Rongzhen Zhang, Hongbo Liang, Xiao-Dong Gao, Hideki Nakanishi, Yan Xu

**Affiliations:** 1 College of Science of China Pharmaceutical University, Nanjing, 211198 People’s Republic of China; 2 Key Laboratory of Industrial Biotechnology of Ministry of Education & School of Biotechnology, Jiangnan University, Wuxi, 214122 People’s Republic of China; 3 Key Laboratory of Carbohydrate Chemistry and Biotechnology, Ministry of Education, School of Biotechnology, Jiangnan University, Wuxi, China; 4 Present Address: School of Biotechnology, Jiangnan University, 1800 Lihu Avenue, Wuxi, 214122 People’s Republic of China

**Keywords:** (*S*)-carbonyl reductase II, Glucose dehydrogenase, Sustainable enantioselective catalysis, Organic solvent, Spore-microencapsulation

## Abstract

**Background:**

*Saccharomyces cerevisiae* AN120 *osw2∆* spores were used as a host with good resistance to unfavorable environment. This work was undertaken to develop a new yeast spore-encapsulation of *Candida parapsilosis* Glu228Ser/(*S*)-carbonyl reductase II and *Bacillus* sp. YX-1 glucose dehydrogenase for efficient chiral synthesis in organic solvents.

**Results:**

The spore microencapsulation of E228S/SCR II and GDH in *S. cerevisiae* AN120 *osw*2∆ catalyzed (*R*)-phenylethanol in a good yield with an excellent enantioselectivity (up to 99%) within 4 h. It presented good resistance and catalytic functions under extreme temperature and pH conditions. The encapsulation produced several chiral products with over 70% yield and over 99% enantioselectivity in ethyl acetate after being recycled for 4–6 times. It increased substrate concentration over threefold and reduced the reaction time two to threefolds compared to the recombinant *Escherichia coli* containing E228S and glucose dehydrogenase.

**Conclusions:**

This work first described sustainable enantioselective synthesis without exogenous cofactors in organic solvents using yeast spore-microencapsulation of coupled alcohol dehydrogenases.
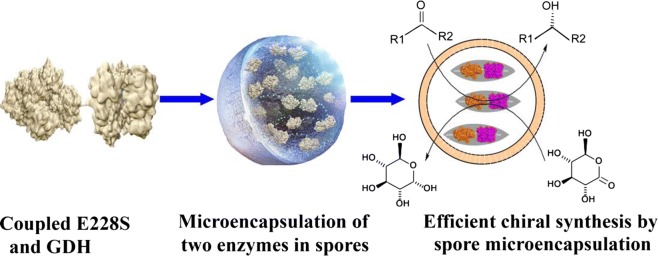

**Electronic supplementary material:**

The online version of this article (10.1186/s12934-019-1137-6) contains supplementary material, which is available to authorized users.

## Background

Alcohol dehydrogenases (ADHs) in organic synthesis have attracted particular interest for the improvement of substrate permeability, cofactor regeneration and process simplification [1, 2]. However, few ADH exhibited solvent-resistant properties associated with their sustainable enantioselective catalytic functions [3]. Enzyme encapsulation is a practical technique to improve enzyme stability and performance, such as the improvement of substrate transportation and product extraction efficiency in organic solvent [4, 5].

To realize efficient enantioselective synthesis, chiral-forming ADH and cofactor-recycling ADH are encapsulated for in situ cofactor regeneration [6, 7]. Because of the complexity and variety of ADHs, co-encapsulation of multiple ADHs in one cage often leads to rapid denaturation and/or sharply decreased activity. Liu et al. encapsulated glutamate dehydrogenase and lactate dehydrogenase and their cofactor in three nanoparticles, but they performed reaction with low concentrated substrates [8]. El-Zahab et al. carried out stereospecific reaction with 10 mM glutamate with particle-tethered NADH shuttled between co-immobilized oxidoreductases on nanoporous silica glass [9, 10]. These unsatisfied efficient reactions suffer from low electron transfer efficiencies because of the separate immobilization of ADHs, exposure of the enzymes to toxic substrates, and the limited enzyme conformational transitions [11–13]. Therefore, it is a long-standing challenge in co-encapsulation of ADHs to enhance enzyme stability and reusability for efficient stereoselective synthesis [14].

*Saccharomyces cerevisiae* spore is reported with good resistance to extreme conditions. In absence of nitrogen and the presence of a nonfermentable carbon source, *S. cerevisiae* cells cease vegetative growth and turn to be spores [15]. Yeast spore walls contain dityrosine and chitosan layers, which can encapsulated proteins and protect encapsulated proteins from various environmental stresses, such as digestive enzymes, heat and organic solvents [16, 17]. We recently deleted *osw*2∆ gene in *S. cerevisiae* AN120 spores, whose dityrosine and chitosan layers are structurally loose with better permeability to substrates [18, 19]. The entrapped enzymes in *S. cerevisiae* AN120 *osw*2∆ spores showed a higher activity than in the vegetative cells [18].

Previously, we reported a mutant of *Candida parapsilosis* (*S*)-carbonyl reductase II (SCR II, EC 1.1.1.148), in which glutamate-228 was replaced with serine (E228S/SCRII). E228S/SCRII and glucose dehydrogenase (GDH, EC 1.1.1.47) from *Bacillus* sp. YX-1 were coexpressed in *Escherichia coli*. The enzyme-coupled system catalyzed acetophenone (AP) to (*R*)-phenylethanol ((*R*)-PE) with an optical purity of 99.5% and a yield of 92.2% within 12 h [3]. It is necessary to improve yield and shorten the reaction duration obtained with *E. coli*. The improvement of substrate solubility and activity under organic solvents are more important for efficient reaction.

In this work, we coencapsulate E228S/SCR II and GDH in *S. cerevisiae* AN120 *osw*2∆. The encapsulated enzymes were entrapped in loose-walled *S. cerevisiae* AN120 *osw2*Δ spores, and the substrates and cofactor passed through the yeast spore smoothly. The spore microencapsulation could catalyze highly concentrated substrate in organic solvents, and exhibited excellent catalytic efficiency with long-term and good recycling stabilities. This work performed efficient enantioselective synthesis in organic solvents using spore-microencapulation containing chiral-forming ADH and cofactor-recycling ADH.

## Results

### Co-encapsulation of E228S/SCRII and GDH in spores of *S. cerevisiae* AN120 osw2Δ

**Fig. 1:**
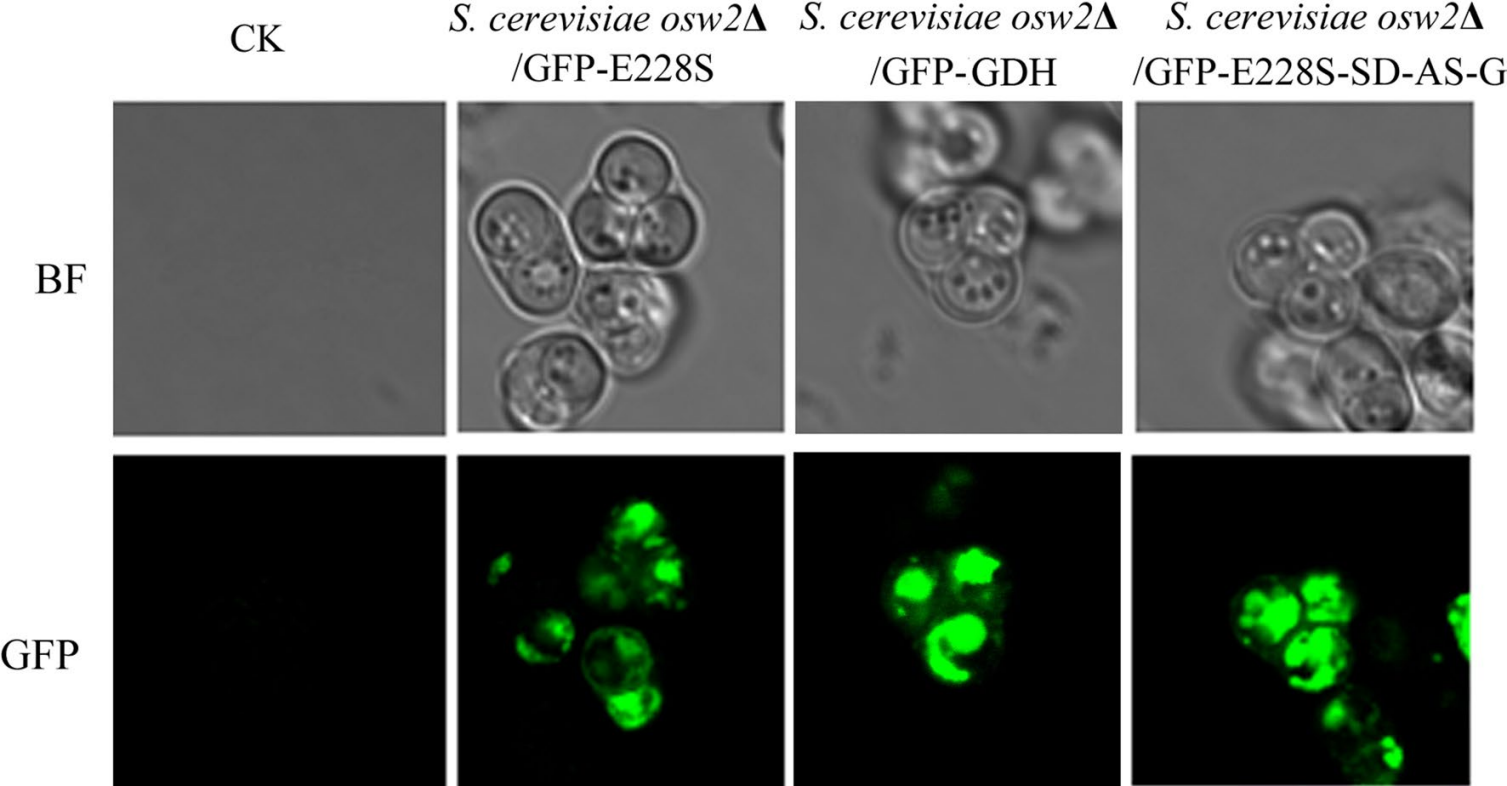
Observation of the green fluorescence distribution in *S. cerevisiae osw2*Δ/GFP-E228S-SD-AS-G. GFP-E228S-SD-AS-G expressed in *S. cerevisiae* AN120 *osw*2∆ cells were sporulated and intact asci were observed under the fluorescent or bright-field (BF) microscopy (×400). Images of *S. cerevisiae osw2*Δ spores with the plasmid pRS424-*TEF*_*pr*_ are shown as a control (CK)

It was reported that the Shine-Dalgarno (SD) and aligned spacing (AS) sequence could initiate protein translation efficiently [20]. The coupled system E228S-SD-AS-G was constructed using SD and AS sequence as a linker between E228S/SCRII and GDH [3]. A monomeric green fluorescent protein (GFP) was fused at N-terminal of E228S-SD-AS-G to construct GFP-E228S-SD-AS-G. The co-encapsulation of E228S/SCRII and GDH in *S. cerevisiae* AN120 *osw2*Δ was performed by SD-Trp screening as described in “Materials and methods” [18]. The protein expression of E228S/SCRII and GDH was confirmed by laser scanning confocal microscope [21]. The green fluorescence distribution in *S. cerevisiae osw2*Δ/GFP-E228S-SD-AS-G cells under laser scanning confocal microscope indicated that most of E228S-SD-AS-G proteins were entrapped in yeast-spore wall or on membrane surface (Fig. 1).

**Fig. 2:**
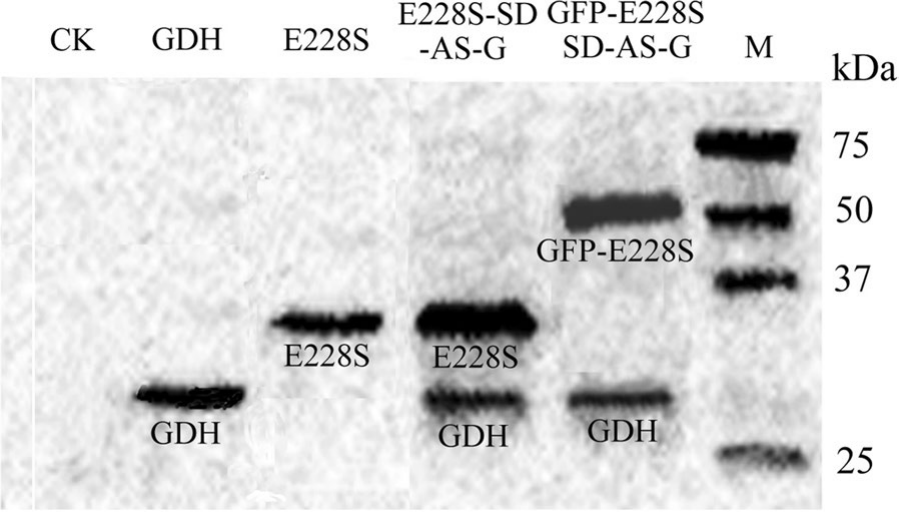
Western blotting analysis of the lysates of the recombinant *S. cerevisiae osw2*Δ spores. The spore with pRS424-*TEF*_*pr*_ plasmid was used as control

The protein expression of E228S/SCRII and GDH was further confirmed by western blotting. A band of 30 kDa was detected in *S. cerevisiae osw2*Δ/E228S spores, which was consistent with molecular mass of E228S. Two bands of 30 kDa and 28 kDa in accordance with E228S and E228S-SD-AS-G were observed in *S. cerevisiae osw2*Δ/E228S-SD-AS-G spores. Since GFP was coexpressed with E228S-SD-AS-G, there were two bands of 57 kDa and 28 kDa (Fig. 2), in accordance with the theoretical molecular mass of GFP-E228S and GDH in *S. cerevisiae osw2*Δ/GFP-E228S-SD-AS-G spores. Those results further suggested E228S/SCRII or/and GDH were expressed and retained inside *S. cerevisiae* AN120 *osw2*Δ spores.

The recombinant *S. cerevisiae osw2*Δ/E228S, *S. cerevisiae osw2*Δ/E228S spore, *S. cerevisiae osw2*Δ/E228S-SD-AS-G and *S. cerevisiae osw2*Δ/E228S-SD-AS-G was dealed with β-glucanase and lyzed by high pressures to obtain their cell-free extracts according to ”Materials and methods”. They showed the specific activity of 46.7, 47.5, 30.5 and 31.1 U mg^−1^ in their cell-free extracts, respectively. By kinetic analysis, the spore-encapsulation *S. cerevisiae osw2*Δ/E228S-SD-AS-G showed the higher *k*_cat_ value (18.9 S^−1^) than the free enzyme E228S-SD-AS-G (15.6 S^−1^), but maintaining a *k*_cat_ value (3.2 μM) essentially the same with respect to the free enzyme.

### Efficient and sustainable (*R*)-PE synthesis by spore microencapsulation in aqueous phase

The free enzyme E228S/SCRII (Free_E228S_), free coupled enzymes containing E228S/SCRII and GDH (Free_E228S-GDH_), *S. cerevisiae osw2*Δ/E228S spore (Spore_E228S_), and *S. cerevisiae osw2*Δ/E228S-SD-AS-G spore (Spore_E228S-GDH_) were used to catalyze 8 g L^−1^ (≈ 67 mM) AP to (*R*)-PE in PBS buffer. Free_E228S_, Free_E228S-GDH_, Spore_E228S_ and Spore_E228S-GDH_ retained 89–95% of yields and over 99% of enantiomeric excess (*ee*) in (*R*)-PE production (Table 1). Spore_E228S_ and Spore_E228S-GDH_ gave the same enantiopurity and slightly lower yield with respect to Free_E228S_ and Free_E228S-GDH_ (Table 1). The spore microencapsulation accomplished the enantioselective synthesis of (*R*)-PE much more quickly than Free_E228S_ and Free_E228S-GDH_ in aqueous phase. The Spore_E228S_ and Spore_E228S-GDH_ (with GDH enzyme for cofactor regeneration) gave 83% and 86% in yields, and over 99% retention in optical purity of (*R*)-PE even if they were reused 10 times for asymmetric biosynthesis (Table 1).

**Table 1:** Enantioselective synthesis of (*R*)-PE by yeast-spore coencapsulation of ADHs and free enzymes in aqueous medium

	Samples	Yield (%)^a^	*ee* (%)^b^	Reduction durations (h)^c^	Productivity (mM h^−1^ g^−1^)
E228S SCRII	Free	93	> 99	8	77.9
	Encapsulated^d^	89	> 99	6	99.4
	Encapsulated (10)	83	> 99	60	92.7
E228S-GDH^e^	Free	95	> 99	6	106.1
	Co-encapsulated	93	> 99	2	311.6
	Co-encapsulated (10)	86	> 99	20	288.1

All reactions were carried out at 30 °C in 100 mM PBS buffer solution (pH 7.4) by using free ADHs (0.1 mg) and Spore_E228S_ or Spore_E228S-GDH_ (0.1 g). The concentration of AP was 8 g/L (≈ 67 mM)

^a^% yield of (*R*)-PE was determined by HPLC

^b^*ee*: enantiomeric excess, the *ee* values of (*R*)-PE were determined using a chiral stationary phase on a Chiralcel OB-H column [3]

^c^Reaction duration was determined when the product (*R*)-PE was produced with the highest yield

^d^The value in parentheses denotes the times the encapsulated enzymes was recycled for reactions

### Spore microencapsulation presents good resistance to extreme conditions

**Fig. 3:**
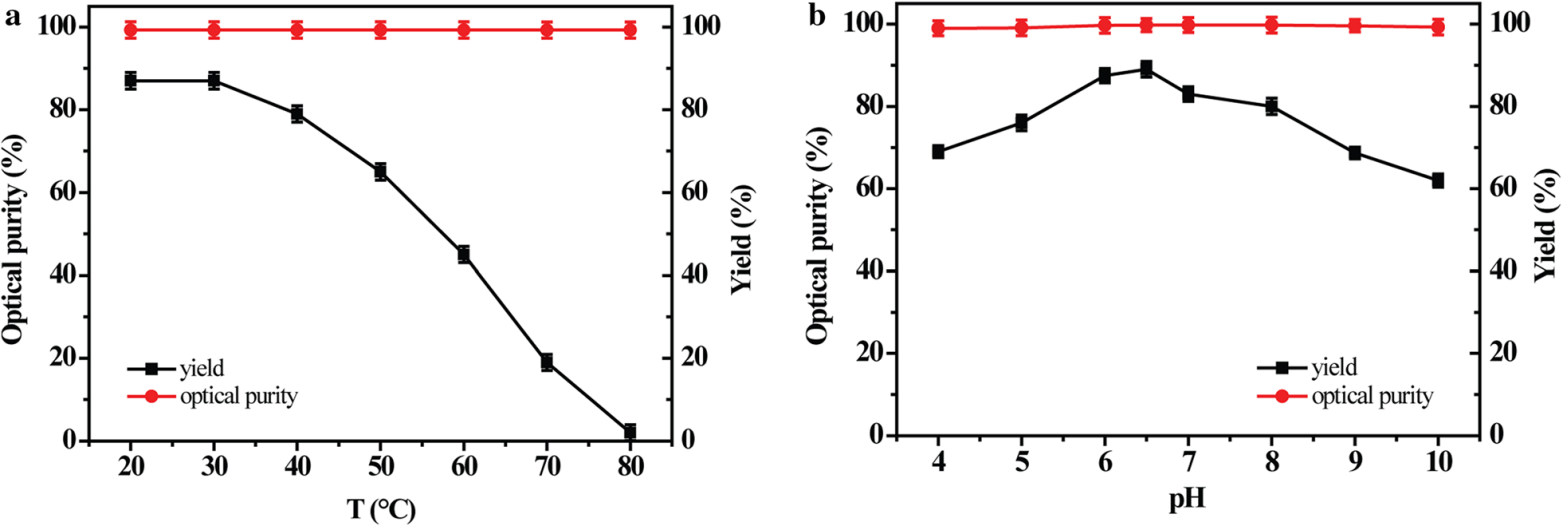
The temperature (**a**) and pH (**b**) dependence of Spore_E228S-GDH_ for asymmetric synthesis of (*R*)-PE. The Spore_E228S-GDH_ was incubated in a buffer containing 100 mM potassium phosphate, pH 6.5 at 20–80 °C for 1 h. The Spore_E228S-GDH_ was incubated between pH 4.0 and 10.0 at 4 °C for 5 h

The Spore_E228S-GDH_ displays similar optimal conditions: 35 °C and pH 6.5 for enantioselective synthesis of (*R*)-PE. The stability of Spore_E228S-GDH_ for asymmetric synthesis of 67 mM AP to (*R*)-PE was investigated under extreme temperature and pH, repetitive freezing–thawing and air-drying conditions. When the Spore_E228S-GDH_ was kept at 20–50 °C for 1 h, it produced (*R*)-PE with 75–93% yield (Fig. 3). It catalyzed the stereoselective synthesis of (*R*)-PE with about 68% yield when the Spore_E228S-GDH_ was incubated at 50 °C for 1 h. When kept at pH 5.0–9.0 for 5 h, the Spore_E228S-GDH_ produced (*R*)-PE with over 75% yield. Even at extreme pH 4.0 and 10.0 for 5 h, it produced (*R*)-PE in yields of over 65%. In all above cases, the Spore_E228S-GDH_ retained excellent optical purity over 99%.

**Fig. 4:**
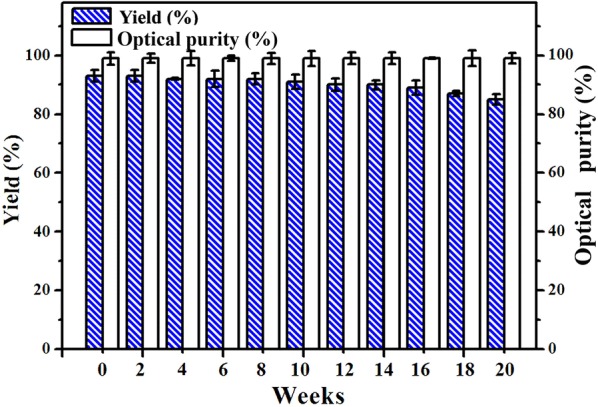
Asymmetric synthesis of (*R*)-PE by Spore_E228S-GDH_ in freezing and thawing cycles. The Spore_E228S-GDH_ was frozen at − 20 °C and was thawed at room temperature. The freezing–thawing process was repeated at least once a day. Some of Spore_E228S-GDH_ was used for enantiospecific reaction, and the rest was taken back at − 20 °C

**Fig. 5:**
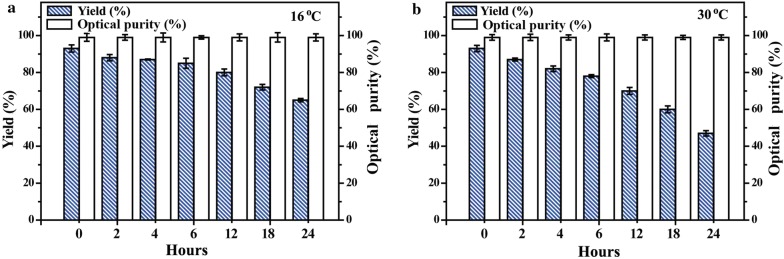
Asymmetric synthesis of (*R*)-PE by Spore_E228S-GDH_ at 16 °C (**a**) and 30 °C (**b**) for air-drying. The Spore_E228S-GDH_ sample (0.1 g) containing encapsulated E228S/SCRII-GDH was stored at 16 °C or 30 °C for air-drying. The samples were taken for asymmetric synthesis of (*R*)-PE each 2–6 h

In repetitive freezing–thawing cycles, virtually no less in optical purity and over 84% yield of (*R*)-PE was detected after 20 weeks (Fig. 4). When the Spore_E228S-GDH_ was air-dried at 16 °C for 12 h and 24 h, it exhibited good yield (82% and 66%) for stereoselective synthesis of (*R*)-PE in PBS solution. When the Spore_E228S-GDH_ was incubated at 30 °C in air-drying conditions for 12 h and 24 h, it produced (*R*)-PE with 69% and 45% yields. In all cases (*R*)-PE was synthesized with over 98% enantioselectivity (Fig. 5).

### Sustainable enantioselective synthesis of (*R*)-PE by spore microencapsulation in organic solvents

**Fig. 6:**
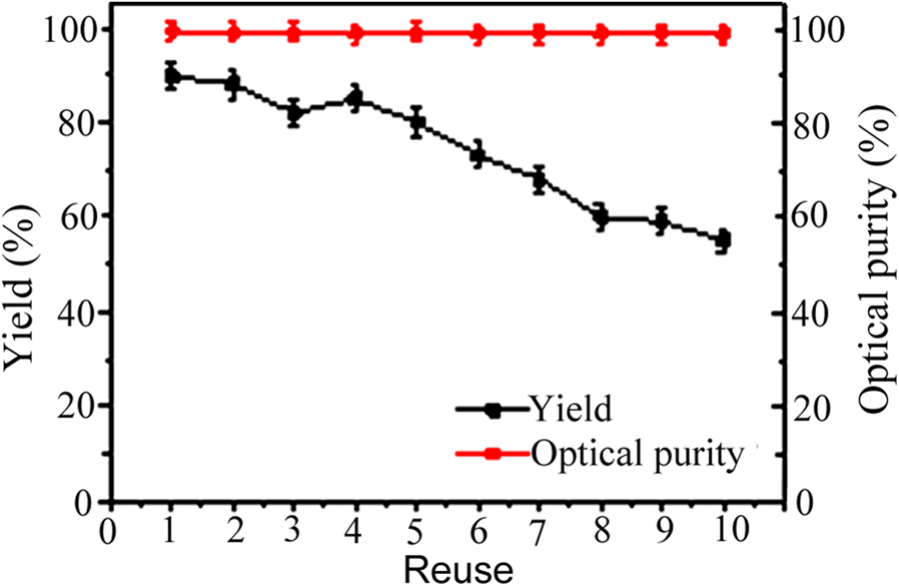
The reusability of spore-microencapsulation Spore_E228S-GDH_ for asymmetric synthesis of (*R*)-PE in ethyl acetate

The enantioselective synthesis of 25 g L^−1^ (≈ 208 mM) AP to (*R*)-PE was carried out by the Spore_E228S-GDH_ in solvent solutions (PBS buffer solution/solvent (170:30 v/v)). The Spore_E228S-GDH_ produces (*R*)-PE in moderate to good yield (51–88%) in solvent solutions (Table 2). When the Spore_E228S-GDH_ were reused for asymmetric reduction in ethyl acetate solution for 6 times, it gave 72% yield and perfect enantioselectivity (> 99% *ee*). The Spore_E228S-GDH_ was reused for (*R*)-PE production for 3–5 times in DMSO, 2-propanol, hexane and urea, it gave over 70% yield and high enantioselectivity (> 93% *ee*) (Table 2). These results indicate that the encapsulation of ADHs in yeast spores is not inactivated by polar solvents, but keep good performance for asymmetric synthesis of AP to (*R*)-PE. Although the Spore_E228S-GDH_ catalyzed (*R*)-PE with a higher yield in the aqueous medium, the use of organic solvent makes the process more efficient by allowing high concentration of substrates (25 g L^−1^ ≈ 208 mM) to be used, and the same yeast-encapsulation can be reused at least six times for sustainable reductions in ethyl acetate (Fig. 6). The use of ethyl acetate makes the Spore_E228S-GDH_ catalyze the enantioselective reaction accessible to hydrophobic substrates. Since the reaction was performed in 15% ethyl acetate and chiral product was extracted with twofold volume of ethyl acetate, more ethyl acetate was added directly to the reaction mixture and simplified the product extraction process after reaction finished.

**Table 2:** Enantioselective synthesis of (*R*)-PE by Spore_E228S-GDH_ in organic solvents

Solvent	Log P	Yield (%)^a^	*ee* (%)	Reduction durations	Productivity (mM h^−1^ g^−1^)
PBS		93 (95)	> 99 (> 99)	4.5 h	429.9 (439.1)
Ethyl acetate	0.68	88 (43)	> 99 (70)	4.5 h (10 h)	406.8 (89.4)
Ethyl acetate (6)^b^	0.68	72	> 99	27 h	554.7
DMSO	− 1.3	81 (46)	> 99 (85)	4 h (10 h)	421.2 (95.7)
DMSO (3)	− 1.3	70	> 99	12 h	121.3
2-Propanol	0.33	85	98	5 h	353.6
2-Propanol (5)	0.33	70	95	25 h	291.2
Tert-butanol	0.6	72 (41)	96 (82)	5 h (12 h)	299.5 (71.1)
Methylbenzene	2.5	70	98	5 h	291.2
Cyclohexane	3.2	51 (39)	98 (92)	5 h (12 h)	212.2 (67.6)
Nonane	2.9	72	96	5 h	299.5
Hexane	3.5	80	98	4.5 h	369.8
Hexane (3)	3.5	70	93	13.5 h	107.9
Ethyl isovalerate	2.3	68	96	4.5 h	314.3
Urea	− 2.1	82	97	4.5 h	379.0
Urea (4)	− 2.1	70	93	18 h	80.9

Unless otherwise stated, all reactions were performed at 30 °C in 100 mM PBS buffer solution (pH 7.4) by using 0.1 g Spore_E228S-GDH_. The reaction solution (2 mL) consists of 1.7 mL 100 mM PBS buffer (pH 7.4) and 0.3 mL solvents. AP concentration was 25 g/L (≈ 208 mM)

^a^(*R*)-PE synthesis results with free enzymes in 200 mL PBS buffer (100 mM, pH 7.4) are given in parentheses. AP concentration was 8 g/L (≈ 67 mM)

^b^The values in parentheses in this column denote the recycled times for enantioselective synthesis

### Enantioselective synthesis of chiral products in ethyl acetate

A serial of phenyl-ring-containing ketones were stereoselectively catalyzed using glucose as a co-substrate by the Spore_E228S-GDH_ in ethyl acetate solution. All reactions were performed with 200 mM substrate. The Spore_E228S-GDH_ catalyzed the enantioselective reaction of AP (**1a**) and 4′-methoxyacetophenone (**3a**) to (*R*)-PE ((*R*)-**1b**) and (*R*)-1-(4-methoxyphenyl ethanol ((*R*)-**3b**) with slightly lower yield in ethyl acetate than free enzyme did in aqueous medium (Table 3). 4′-Methylacetophenone (**2a**), 4′-bromoacetophenone (**4a**) and 4′-chloroacetophenone (**5a**) were catalyzed to their corresponding chiral alcohols ((*R*)-**2b**, (*R*)-**4b** and (*R*)-**5b**) with high yields (79–85%) and excellent enantioselectivity (> 99% *ee*) by the Spore_E228S-GDH_ in ethyl acetate (Table 3), with a little lower yield in ethyl acetate but the similar enantioselectivity when compared with the same resulting products by the free enzyme in PBS buffer (Table 3). 2-Chloroacetophenone (**6a**) and 3-chloroacetophenone (**7a**) were catalyzed to (*R*)-1-(2-chlorophenyl) ethanol ((*R*)-**6b**) and (*R*)-1-(3-chlorophenyl) ethanol ((*R*)-**7b**) with almost the same yields by using Spore_E228S-GDH_ in ethyl acetate comparable with free enzymes in aqueous medium (Table 3).

**Table 3:** Enantioselective reaction of phenyl-ring-containing ketones and keto esters by Spore_E228S-GDH_ in ethyl acetate solution

*n*	R1	R2	Abs.Config.^a^	Yield (%)	*ee* (%)	Time (h)
1	Ph	CH_3_	(*R*)-1b	88 (93)^b^	>99 (> 99)	4.5 (10)
2	Ph-*p*-CH_3_	CH_3_	(*R*)-2b	79 (89)	>99 (> 99)	4 (9)
3	Ph-*p*-OCH_3_	CH_3_	(*R*)-3b	45 (51)	>99 (> 99)	4 (10)
4	Ph-*p*-Br	CH_3_	(*R*)-7b	81 (90)	>99 (> 99)	4 (10)
5	Ph-*p*-Cl	CH_3_	(*R*)-6b	85 (93)	>99 (> 99)	4 (10)
6	Ph-*o*-Cl	CH_3_	(*R*)-4b	20 (17)	>99 (> 99)	4 (10)
7	Ph-*m*-Cl	CH_3_	(*R*)-5b	25 (28)	>99 (> 99)	4 (10)
8	Ph	CH_2_OH	(*S*)-8b	14 (13)	>99 (> 99)	4 (8)
9	CH_3_(CH_2_)_2_	CH_3_	(*R*)-9b	60 (34)	94 (86)	3 (8)
10	CH_3_(CH_2_)_3_	CH_3_	(*R*)-10b	56 (27)	91 (73)	3 (8)
11	CH_3_(CH_2_)_4_	CH_3_	(*R*)-11b	53 (22)	89 (68)	3 (8)
12	CH_3_(CH_2_)_5_	CH_3_	(*R*)-12b	43 (19)	85 (65)	3 (8)
13	CH_3_COO	O-CH_3_	(*R*)-13b	55 (35)	97 (83)	5 (12)
14	CCl_3_COCH_2_	O-CH_2_CH_3_	(*R*)-14b	52 (26)	90 (77)	5 (12)
15	Ph-CO(CH_2_)_2_	O-CH_3_	(*R*)-15b	64 (40)	95 (80)	5 (12)

Unless otherwise stated, all reactions were performed at 30 °C in 1.7 mL PBS buffer (100 mM, pH 7.4) and 0.3 mL ethyl acetate by using 0.1 g Spore_E228S-GDH_. The substrate concentration was 200 mM

^a^Absolute configuration. It was determined by comparing the retention time with that of standard samples

^b^The values in parentheses denote the enantioselective synthesis results of 60 mM substrate to the corresponding chiral products with free enzyme in 2.0 mL PBS buffer (100 mM, pH 7.4) at 30 °C

Although the linear chain ketones, such as 2-hexanone (**9a**), 2-pentanone (**10a**), 2-heptanone (**11a**) and 2-octanone (**12a**) were catalyzed to (*R*)-2-hexanol ((*R*)-**9b**), (*R*)-2-pentanol ((*R*)-**10b**), (*R*)-2-heptanol ((*R*)-**11b**) and (*R*)-2-octanol ((*R*)-**12b**) with moderate enantioselectivity (65–86% *ee*) and a rather low yield (19–34%) by using free enzyme in aqueous medium, significantly improved yields (43–60%) and optical purity (85–94%) were obtained by using the Spore_E228S-GDH_ in ethyl acetate (Table 3). The enantioselective reaction of sterically demanding keto esters was chosen for asymmetric synthesis test by the Spore_E228S-GDH_ in ethyl acetate. Methyl acetoacetate (**13a**), ethyl benzoylacetate (**14a**) and ethyl 4,4,4-trichloroacetoacetate (**15a**) were transformed to (*R*)-methyl-3-hydroxybutyrate ((*R*)-**13b**), (*R*)-ethyl-3-hydroxy-3-phenylpropionate ((*R*)-**14b**) and (*R*)-ethyl-3-hydroxy-4,4,4-trichlorobutyrate ((*R*)-**15b**) with moderate enantioselectivity (77–83%) and rather low yields (26–40%) using free enzymes in PBS solution. Interestingly, the yields of (*R*)-**13b**, (*R*)-**14b** and (*R*)-**15b** were significantly increased and their *ee* values were improved to 90–97% by using the Spore_E228S-GDH_ in ethyl acetate (Table 3).

## Discussion

We previously constructed a coupled system E228S-SD-AS-G using a Shine–Dalgarno (SD) and aligned spacing (AS) sequence as a linker between E228S/SCRII and GDH [3]. In this work, we coexpressed E228S/SCRII and GDH in *S. cerevisiae* AN120 *osw2∆*. With potassium acetate as the only carbon, *S. cerevisiae* cells cease vegetative growth and turn to be spores [15], so the two enzymes: E228S/SCRII and GDH were encapsulated in the yeast spores. The spore encapsulation was confirmed by laser scanning confocal microscope. Gerke et al. reported many genes involved in aerobic respiration, an essential pathway for sporulation, so the sporulation efficiency varied in diverse yeast strains [22]. When the cells was cultured in 2% potassium acetate medium for 24 h, the ratio between spores and cells was over 9:1 by microscopic examination, the spores were harvested and lyzed by high pressures and the active yeast-spores encapsulated E228S/SCRII and E228S-SD-AS-G, named Spore_E228S-GDH_ were obtained for further experiments.

The yeast cells and its corresponding spore exhibited almost the same catalytic activity. The *S. cerevisiae osw2*Δ/E228S exhibit higher activity than E228S in the coupled system. Suda et al. showed that the outermost dityrosine layer works as a diffusion barrier for soluble proteins [16]. Since *osw*2∆ gene in *S. cerevisiae* AN120 spores was deleted, whose dityrosine and chitosan layers are structurally loose, making substrate enter into or out of the cells more easily. So the entrapped enzymes in *S. cerevisiae* AN120 *osw*2∆ spores did not decreased the enzyme activity [18]. The spore encapsulation resulted in the higher catalytic efficiency (5.87 × 10^6^ S^−1^ M^−1^) than the free enzyme. The reaction catalyzed by the SCRs is a sequential type, with the coenzymes binding to the free form of the enzyme and subsequently to the substrates according to the kinetics of the interaction [23]. The change in *k*_cat_ value may be due to the faster cofactor transfer in the reaction system by spore-encapsulation.

Enantiopure aryl alcohols serve as valuable intermediates for preparation of antidepressants, anti-asthmatics, cholesterol-lowering agents, adrenergic receptor agonists, and NK1 antagonists, etc. [24, 25]. For example, (*R*)-PE is a very useful chiral block in the fine chemical and pharmaceutical industry [26, 27]. The Spore_E228S-GDH_ catalyzes the biosynthesis of AP to (*R*)-PE within 2 h, threefold shorter compared to Free_E228S-GDH_. The recombinant (*S*)-carbonyl reductase in *S. cerevisiae* produced chiral products with an optical purity of 92.3% and a yield of 81.8% in 24 h [28]. The recombinant *E. coli* harboring SCRII and GDH performed enantioselective reaction within 12 h [3]. The significantly decreased reaction time by Spore_E228S-GDH_ than Free_E228S-GDH_ can be partly attributed to the three reasons: the free transportation of substrate and cofactor into and out of *S. cerevisiae* AN120 *osw2*Δ spores [18]; the “pure” conditions in the yeast spores, where few other enzymes, i.e., proteases does not bother the enantioselective reactions [16, 19]; and the assembly of ADH enzymes into cell-molecular architectures (yeast spores) enhancing the catalytic efficiency and/or preventing loss of toxic intermediates that hinder cellular functions [18, 29, 30]. Higher enantioselective efficiency by the Spore_E228S-GDH_ than Spore_E228S_ is due to the introduction of GDH for in situ cofactor regeneration in Spore_E228S-GDH_ [31]. This is consistent with the study of ADHs-catalyzed reactions by Gröger et al., who developed a highly efficient “designer cells” for the desired asymmetric reaction by coexpressing the corresponding alcohol dehydrogenase with GDH enzyme [32].

The Spore_E228S_ and Spore_E228S-GDH_ could be recycled for stereoselective reaction, while the Free_E228S_ and Free_E228S-GDH_ could not be reused since ethyl acetate (required for the product (*R*)-PE extraction) denatured them. The Spore_E228S_ and Spore_E228S-GDH_ gave 83% and 86% in yields, and over 99% retention in optical purity of (*R*)-PE even if they were reused 10 times for asymmetric biosynthesis. It was very necessary to add 40 mM cycloheximide to the reaction mixture to prevent spore germination for each new reaction because cycloheximide became inactivated during turnover [21, 33]. So the yeast spores were easily prepared and maintained for the sustainable biocatalysis.

More importantly, *Saccharomyces cerevisiae* spore has good resistance to extreme conditions. Much higher yields and optical purity by using the Spore_E228S-GDH_ were obtained in ethyl acetate than free enzyme in aqueous phase. Those results are consistent with the TeSADH-catalyzed stereospecificial reduction reported by Phillips’s group [34]. The enhanced yield in the enantioselective catalysis of linear ketones by the Spore_E228S-GDH_ in ethyl acetate than free enzyme in PBS solution may be due to the three reasons: the spore encapsulation supplies ADH enzymes with good resistance in organic solvents [16, 17]; the using of ethyl acetate can improve substrate solubility and accelerate the mass transportation; The different salvation of the enzyme active site by organic solvent makes the binding of substrate in the “wrong” orientation and favourable entropically. Filho et al. thought that the solvent functionality would be much more important rather than a single physicochemical parameter associated with the biocompatibility of organic solvents in ADH-catalyzed in biphasic reactions [35]. The enzyme Spore_E228S-GDH_ exhibited different specificity towards ketones and ketoesters. The steric conformation of the active site in the catalytic domain determines the substrate recognition, binding, and orientation in the enzyme [36]. The enzyme SCRII/E228S followed the anti-Prelog rule for their behaviors in catalyzing asymmetric reduction of 2-HAP. SCRII poorly catalyzes the *ortho* substituted derivatives of acetophenone and 2-HAP with chloro or methyl at various positions of the phenyl ring, suggesting that the substitution at *ortho* position might have a steric effect on the hydrogen attack from electron donator NADPH to the carbonyl group and therefore significantly influence the biotransformation efficiency of the enzymes.

More significantly, the Spore_E228S-GDH_ showed an extremely high long-term stability in repetitive freezing–thawing cycles; virtually no less in optical purity and a high yield of (*R*)-PE was detected after 20 weeks. When the Spore_E228S-GDH_ was air-dried, it exhibited good yield for stereoselective synthesis of (*R*)-PE in PBS solution. These results further confirmed that the spore-encapsulation could protect catalytic functions of entrapped enzymes from extreme conditions [34]. However, the recombinant yeast SCRs was not reported to have the good resistance [28, 37].

We previously reported a coupled system containing *C. parapsilosis* E228S/SCRII and *Bacillus* sp. YX-1 GDH catalyzes the reduction of AP to (*R*)-PE, but the higher substrate concentrations, shorter reaction time and sustainable reaction in organic solvents are preferred [3]. Liposomal encapsulation is reported as one of the best methods to protect enzymes against unsuitable external environments for stabilization of the enzyme structure and activity, but the low substrate permeability across the lipid membrane drastically limits the turnovers of encapsulated enzymes and thus has rather limited application [38–40]. Nasseau et al. improved the substrate permeability through the liposomes to a certain extent using a channel Omp F from the outer cell wall of *E. coli*, but they did not use it in enantioselective synthesis [41]. In 2007, Phillips’s group successfully encapsulated Trp110Ala secondary alcohol dehydrogenase in xerogel for efficient reduction of hydrophobic ketones in organic solvents [34]. They reported a single-batch asymmetric reduction in water-immiscible organic solvents but rather the sustainable enantioselective synthesis using reusable catalysts would be of great significance [34]. The co-encapsulation of E228S/SCRII and GDH in *S. cerevisiae* AN120 *osw2*Δ spore presented good resistance under extreme conditions and improved permeability of substrate/cofactor. During the interaction between enzymes and cofactors/the precursors, the active enzymes, E228S/SCRII and GDH were entrapped in loose-walled *S. cerevisiae* AN120 *osw2*Δ spores, and the cofactor and precursors passed through the yeast spore walls smoothly [42, 43]. Moreover, the enzyme in spores showing good resistance and ability might be due to the enzyme in spores preventing it from protein denaturation by solvents.

The use of spore-microencapsulation of ADHs is of great advantage for several reasons beside the very economical and straightforward process operation (only the spore-encapsulated catalyst (as wet biomass), substrate, glucose, and a very small amount of cofactor were needed to start the reaction). First, it is much more stable than the free form, which makes it more suitable for variable reaction conditions, and more attractive to synthesis in organic solvents. Second, it makes the ADH enzymes with long-term reusability. Third, it makes enantioselective reaction proceed with easy product separation process since asymmetric reaction was performed in ethyl acetate.

## Conclusion

In summary, we have developed a novel microencapsulation technique using yeast spore to coencapsulate *C. parapsilosis* E228S/SCRII and *Bacillus* sp. YX-1 GDH. The encapsulation Spore_E228S-GDH_ shows good resistance to extreme conditions and excellent catalytic function with long-term and good recycling stabilities even with highly concentrated substrate. To the best of our knowledge, this work first described an efficient enantioselective synthesis in organic chemicals using spore coencapsulation of chiral-forming ADH and cofactor-recycling ADH.

## Materials and methods

### Microorganisms

*Candida parapsilosis* Glu228Ser/(*S*)-carbonyl reductase (E228S/SCR II, EC1.1.1.148) and *Bacillus* sp. YX-1 glucose dehydrogenase (GDH, EC1.1.1.47) were obtained as described previously [3]. *Escherichia coli* JM109 was used as the host for gene cloning experiments. *E*. *coli* BL21/E228S and *E*. *coli*/E228S-SD-AS-G was used as the DNA donor [3]. *Saccharomyces cerevisiae* AN120 *osw*2∆ whose *osw*2∆ gene was knocked out was used as the host for gene expression. *E*. *coli* was cultured at 37 °C in Luria–Bertani (LB) medium, supplemented with kanamycin (50 μg mL^−1^) when necessary. *S. cerevisiae* was cultured at 30 °C in yeast extract peptone dextrose (YPD) medium overnight, then 10 mL of the culture was shifted to 1 L YPAcetate (1% yeast extract, 2% peptone, 2% potassium acetate) and grown 24 h. The yeast cells were harvested by centrifugation and resuspended in 1 L of 2% potassium acetate medium, and cultured for 24 h to obtain spores.

### Chemicals

Acetophenone (AP) (98%), 4′-methylacetophenone (98%), 4′-methoxyacetophenone (98%), 4′-bromoacetophenone (98%), 2′-chloroacetophenone (97%), 3′-chloroacetophenone (98%), 4′-chloroacetophenone (98%), 2-hydroxyacetophenone (99%), 2-pentanone (99%), 2-hexanone (99%), 2-heptanone (99%), 2-octanone (99%), methylacetoacetate (99%), ethyl 4,4,4-trichloroacetoacetate (99%), ethylbenzoylacetate (99%), their corresponding chiral products, and NADPH were purchased from the Sigma-Aldrich Chemical Co. Inc (Shanghai, China) or obtained commercially. All other chemicals were of the highest grade that could be obtained commercially.

### Construction of coupled E228S/SCRII and GDH in S. cerevisiae AN120 osw2∆

Since the recombinant *E. coli* coexpressing Glu228Ser and GDH needed 12 h to complete asymmetric reaction, *S. cerevisiae osw2*Δ was attempted to use for their expression to shorten reaction duration. E228S-SD-AS-G contains E228S/SCRII (SCRII Genbank ID: GQ411433) [3] and GDH genes (Genbank ID: NC014551.1) with a Shine-Dalgarno (SD) and aligned spacing (AS) sequence between them. The sequence of SD-AS is GAAGGAGATATACC with the supplemental amino acids of Arg-Arg-Asp-Ile. The 6× Histine was fused with N teminal of E228S and C terminal of GDH. The *Pst* I/*Xho* l fragments (E228S, GDH and E228S-SD-AS-G) were inserted into the *Pst* I/*Xho* l site of pRS424-*TEF*_*pr*_ and pRS424-*TEF*_*pr*_-GFP plasmid, respectively. The resulting plasmids pRS424-*TEF*_*pr*_-E228S, pRS424-*TEF*_*pr*_-GDH, pRS424-*TEF*_*pr*_-E228S-SD-AS-G pRS424-*TEF*_*pr*_-GFP-E228S, pRS424-*TEF*_*pr*_-GFP-GDH and pRS424-*TEF*_*pr*_-GFP-E228S -SD-AS-G were confirmed by DNA sequencing.

The six recombinant plasmids were chemically introduced into *S. cerevisiae* AN120 *osw*2∆ competent cells respectively. The positive strains *S. cerevisiae osw2*Δ/E228S, *S. cerevisiae osw2*Δ/GDH, *S. cerevisiae osw2*Δ/E228S-SD-AS-G, *S. cerevisiae osw2*Δ/GFP-E228S, *S. cerevisiae osw2*Δ/GFP-GDH and *S. cerevisiae osw2*Δ/GFP-E228S-SD-AS-G were obtained by SD-Trp screening [18].

### Obtaining spore-microencapsulated ADHs

The spore-encapsulation was prepared using the method as described by Kloimwieder and Winston [44] with some minor modification. The above six recombinant *S. cerevisiae osw2*Δ were grown in YPD liquid media with appropriate supplemental amino acids overnight, and then shifted to the medium containing 1% yeast extract, 2% peptone and 2% potassium acetate for 24 h. The cells were harvested by centrifugation, washed with H_2_O, resuspended in 30 mL of 2% potassium acetate medium, and cultured for 24 h, when sporulation efficiency (the number ratio of spores and cells) of *S. cerevisiae osw2*Δ was over 90% by microscopy [22]. The spores resuspended in 1 mL spheroplast buffer (pH 7.0) containing 50 mM potassium phosphate, 1.4 M sorbitol, 40 mM β-mercaptoethanol, and mixed with 50 µL β-glucanase solution (1 mg β-glucanase was dissolved in 500 µL of 50% glycerol). After 1 h of incubation at 37 °C, spores were washed twice with spheroplast buffer, and then resuspended in spheroplast buffer and sonicated to disrupt the ascal membrane [44]. The resulting pellet were washed three times with 0.5% Triton-X and resuspended in 1 mL of 0.5% Triton-X and layered on top of Percoll (Sigma-Aldrich, Shanghai, China) gradients (50–80% Percoll, 10% 2.5 M sucrose and 0.5% Triton-X). After centrifugation at 15,000×*g* at 4 °C for 1 h, the vegetative cells and debris was removed. The purified spore encapsulation containing E228S/SCRII and GDH were obtained and stored at − 20 °C.

### Microscopy

To indentify the spores keeping in dormancy, the microscope images of spores were observed by using Nikon Eclipse Ti-E inverted microscope equipped with DS-Ri1 camera and NIS-Element AR software (Nikon, Tokyo, Japan). Samples were drop-cast from sodium phosphate buffer solution onto copper coated TEM grids. Transmission electron microscopy was carried out on a JEOL 2000EX operating at 200 keV. The spore and cell numbers were observed and counted in 20 visual fields by microscopy. The number ratio between spore and cells was calculated. The average value of ratio from visual fields was defined as sporulation efficiency [18].

### Western blotting

Western blot analysis of spores was performed to indentify the ADH expression in *S. cerevisiae* AN120 *osw2*Δ spores. Spores were washed with water, suspended in 500 μL of 8 M urea, and lysed by high pressure for 1 h on ice. The ADH specificity in the total proteins was probed with His × tag monoclonal antibody (Ab1, Novagen), followed by rabbit anti-mouse IgG conjugated to horseradish peroxidase (Ab2, BioLab) against Ab1 and visualized by Enhanced Chemiluminescence system (Amersham) for 15 min according to the instructions.

### Enzyme assay and kinetics measurement

The enzymatic activities of E228S/SCRII for the reduction of AP were measured at 35 °C by recording the rate of change in the NAD(P)H absorbance at 340 nm [45]. The assay mixture for the GDH activity contained 100 mM Tris–HCl (pH 8.0), 100 mM glucose, and 2 mM NADP^+^, and the reactions at 30 °C. One unit of activity is defined as the amount of enzyme catalyzing the oxidation of 1 μmol NADPH or the reduction of 1 μmol NAD(P)^+^ under the measurement condition, respectively.

The kinetic parameters of protein were measured and calculated using a Beckman DU-7500 spectrophotometer with a Multicomponent/SCA/Kinetics Plus software package and a thermostated circulating water bath. Substrate (0.5 to 20 mM), enzyme (~ 30 μM, 1 mg mL^−1^), and cofactor NADPH (5 mM) in 100 mM potassium phosphate buffer (pH 6.0) were used for a series of assays. Each value was calculated depending on three independent measurements and all standard errors of fits were not more than 5%. Kinetic parameters were derived from Michaelis–Menten plots and Lineweaver–Burk.

### Biotransformation and analytical methods

The asymmetric biotransformation by spore-microencapsulation were carried out in a 10 mL or 500 mL flask equipped with a magnetic stirrer as described previously [3], with minor modifications. The reaction mixture consisted of 0.1 M potassium phosphate buffer (pH 6.5), 8–25 g L^−1^ AP, 10–30 g L^−1^ glucose, 0.02 mM NADPH, and 0.1 g yeast spores at 35 °C. Cycloheximide of 40 mM was added in the reaction mixture to prevent spore germination [33]. The reaction time was from 1 to 10 h. At the end of the reaction, the products were extracted with 100% ethyl acetate which was twofold volume of reaction mixture, and the organic layer was used for analysis. The optical purity and yield of the product were determined using HPLC or GS on a Chiralcel OB-H column (Daicel Chemical Ind. Ltd., Japan). The calculation equations are as follows: the optical purity of (*R*)-enantiomer = [(S_R_ − S_S_)/(S_S_ + S_R_)] × 100%, and the yield of (*R*)-enantiomer = (CR/138)/(CH/136). S_R_, the peak area of (*R*)-enantiomer; S_S_, the peak area of (*S*)-enantiomer; CR, peak area corresponding to concentration of (*R*)-enantiomer after reaction; CH, peak area corresponding to concentration of substrate before reaction.

### Stability of spore encapsulation

For thermal inactivation, the spore-microencapsulation Spore_E228S-GDH_ was incubated in 100 mM potassium phosphate (pH 6.5) at 20–80 °C for 1 h [32]. To determine pH dependence, the entrapped enzymes was incubated between pH 4.0 and 10.0 at 4 °C for 5 h. To determine organic resistance, all reaction were performed as described above except with the addition of 15% organic solvents.

For air-drying treatment, the spore microencapsulation was kept in incubator at 16 °C or 30 °C for 24 h. For repetitive freezing–thawing treatment, the spore microencapsulation was frozen at − 20 °C and was thawed at room temperature. The freezing–thawing process was repeated at least once a day. Some of spore microencapsulation was used for reaction, and the rest was taken back to refrigerator at − 20 °C. The experiments lasted for 20 weeks.

### Usability of spore microencapsulation

After each enantioselective synthesis was finished, the spore microencapsulation was washed with H_2_O. After centrifugation, the supernatant was removed and the washed spores were used for next reaction. Cycloheximide (40 mM) was added to the reaction system to prevent spore germination for each new reaction (Additional file 1).

## Additional file

**Additional file 1: Figure S1.** Microscope images of enzyme-encapsulated spores cultured with YPD and reaction mixture. **Figure S2.** Optimal temperature and pH value on enantioselective synthesis of (*R*)-PE by Spore_E228S-GDH_. **Table S1.** Strains and plasmids in this study. **Table S1.** Strains, plasmids and primers in this study. **Table S2.** Analytical methods of corresponding chiral products.

## Data Availability

The datasets of supporting the conclusions in this article are included in the manuscript and additional file.

## Abbreviations

AP: acetophenone
E228S: Glu228Ser
*ee*: enantiomeric excess
Free_E228S_: free enzyme E228S/SCRII
Free_E228S-GDH_: free coupled enzymes containing E228S/SCRII and GDH
GDH: glucose dehydrogenase
(*R*)-PED: (*R*)-phenylethanol
SCRII: (*S*)-carbonyl reductase
Spore_E228S_: *S. cerevisiae osw2*Δ/E228S spore containing E228S
Spore_E228S-GDH_: *S. cerevisiae osw2*Δ/E228S spore containing E228S and GDH

## References

[1] Gröger H, Hummel W, Buchholz S, Drauz K, Nguyen TV, Rollmann C, Hüsken H, Abokitse K (2003). Practical asymmetric enzymatic reduction through discovery of a dehydrogenase-compatible biphasic reaction media. Org Lett.

[2] Wang S, Xu Y, Zhang RZ, Zhang BT, Xiao R (2012). Improvement of (*R*)-carbonyl reductase-mediated biosynthesis of (*R*)-1-phenyl-1,2-ethanediol by a novel dual-cosubstrate-coupled system for NADH recycling. Process Biochem.

[3] Zhang RZ, Zhang BT, Xu Y, Li YH, Li M, Liang HB, Xiao R (2013). Efficicent (*R*)-phenylethanol production with enantioselectivity-alerted (*S*)-carbonyl reductase II and NADPH regeneration. PLoS ONE.

[4] Cao L, Langen LV, Sheldon RA (2003). Immobilised enzymes: carrier-bound or carrier-free?. Curr Opin Biotechnol.

[5] Gervais TR, Carta G, Gainer JL (2010). Asymmetric synthesis with immobilized yeast in organic solvents: equilibrium conversion and effect of reactant partitioning on whole cell biocatalysis. Biotechnol Prog.

[6] Schroer K, Zelic B, Oldiges M, Lütz S (2009). Metabolomics for biotransformations: intracellular redox cofactor analysis and enzyme kinetics offer insight into whole cell processes. Biotechnol Bioeng..

[7] Wandrey C (2004). Biochemical reaction engineering for redox reactions. Chem Rev.

[8] Liu W, Zhang S, Wang P (2009). Nanoparticle-supported muti-enzyme biocatalysis with in situ cofactor regeneration. J Biotechnol.

[9] El-Zahab B, Donnelly D, Wang P (2008). Particle-tethered NADH for production of methanol from CO(2) catalyzed by coimmobilized enzymes. Biotechnol Bioeng.

[10] El-Zahab B, Jia H, Wang P (2004). Enabling multienzyme biocatalysis using nanoporous materials. Biotechnol Bioeng.

[11] Garg B, Bish T, Ling Y-C (2015). Graphene-based nanomaterials as efficient peroxidase mimetic catalysts for biosensing applications: an overview. Molecules.

[12] Hu Q, Kattic PS, Gu Z (2014). Enzyme-responsive nanomaterials for controlled drug delivery. Nanoscale..

[13] Mazurenko I, Monsalve K, Rouhana J, Parent P, Laffon C, Goff AL, Szunerits S, Boukherroub R, Giudici-Orticoni M-T, Mano N, Lojou E (2016). How the intricate interactions between carbon nanotubes and two bilirubin oxidases control direct and mediated O_2_ reduction. ACS Appl Mater Interfaces.

[14] Liu W, Wang P (2007). Cofactor regeneration for sustainable enzymatic biosynthesis. Biotechnol Adv.

[15] Neiman A (2005). Ascospore formation in the yeast *Saccharomyces cerevisiae*. Microbiol Mol Biol Rev.

[16] Suda Y, Rodriguez R, Coluccio A, Neiman A (2009). A screen for spore wall permeability mutants identifies a secreted protease required for proper spore wall assembly. PLoS ONE.

[17] Coluccio A, Bogengruber E, Conrad MN, Dresser ME, Briza P, Neiman AM (2004). Morphogenetic pathway of spore wall assembly in *Saccharomyces cerevisiae*. Eukaryot Cell.

[18] Shi L, Li Z, Tachikawa H, Gao X, Nakanishi H (2014). Microencapsulation of enzymes using yeast spores. Appl Environ Microbiol.

[19] Neiman A (2011). Sporulation in the budding yeast *Saccharomyces cerevisiae*. Genetics.

[20] Chen H, Bjerknes M, Kumar R, Jay E (1994). Determination of the optimal aligned spacing between the Shine-Dalgarno sequence and the translation initiation codon of *Escherichia coli* mRNAs. Nucleic Acids Res.

[21] Tufvesson P, Lima-Ramos J, Nordblad M, Woodley JM (2011). Guidelines and cost analysis for catalyst production in biocatalytic processes. Org Process Res Dev.

[22] Gerke JP, Chen CTL, Cohen BA (2006). Natural isolates of *Saccharomyces cerevisiae* display complex genetic variation in sporulation efficiency. Genetics.

[23] Mu XQ, Xu Y, Yang M, Sun ZH (2006). Steady-state kinetics of the oxidation of (*S*)-1-phenyl-1,2-ethanediol catalyzed by alcohol dehydrogenase from *Candida parapsilosis* CCTCC M203011. J Mol Catal B..

[24] García-Urdiales E, Alfonso I, Gotor V (2011). Update 1 of: enantioselective enzymatic desymmetrizations in organic synthesis. Chem Rev.

[25] Huang Y, Liu N, Wu XR, Chen YJ (2010). Dehydrogenases/reductases for the synthesis of chiral pharmaceutical intermediates. Curr Org Chem.

[26] Kroutil W, Mang H, Edegger K, Faber K (2004). Recent advances in the biocatalytic reduction of ketones and oxidation of sec-alcohols. Curr Opin Chem Biol.

[27] Schmid A, Dordick JS, Hauer B, Kiener A, Wubbolts M, Witholt B (2001). Industrial biocatalysis today and tomorrow. Nature.

[28] Zhang R, Xu Y, Wang S, Zhang B, Geng Y (2011). Expression and subcellular location of (*R*)- and (*S*)-specific carbonyl reductases from *Candida parapsilosis* in *Saccharomyces cerevisia*. Acta Microbiol Sinica.

[29] Yang X, Li Z, Liu B, Klein-Hofmann A, Tian G, Feng Y, Ding Y, Su D, Xiao F (2006). “Fish-in-net” encapsulation of enzymes in macroporous cages as stable, reusable, and active heterogeneous biocatalysts. Adv Mater.

[30] Kang S, Douglas T (2010). Some enzymes just need a space of their own. Science.

[31] Stampfer W, Kosjek B, Moitzi C, Kroutil W, Faber K (2002). For selected contributions on substrate-coupled cofactor regeneration with 2-propanol in the asymmetric enzymatic reduction of ketones. Angew Chem.

[32] Gröger H, Chamouleau F, Orologas N, Rollmann C, Drauz K, Hummel W, Weckbecker A, May O (2006). Enantioselective reduction of ketones with “designer cells” at high substrate concentrations: highly efficient access to functionalized optically active alcohols. Angew Chem Int Ed.

[33] Herman P, Rine J (1997). Yeast spore germination: a requirement for Ras protein activity during re-entry into the cell cycle. EMBO J.

[34] Musa MM, Ziegelmann-Fjeld KI, Vieille C, Zeikus JG, Phillips RS (2010). Xerogel-encapsulated W110A secondary alcohol dehydrogenase from *Thermoanaerobacter ethanolicus* performs asymmetric reduction of hydrophobic ketones in organic solvents. Angew Chem Int Ed.

[35] Filho MV, Stillger T, Müller M, Liese A, Wandrey C (2003). Choice of solvents for biphasic enzymatic reactions?. Angew Chem Int Ed.

[36] Zhu D, Mukherjee C, Rozzell JD, Kambourakis S, Hua L (2006). A recombinant ketoreductase tool-box. Assessing the substrate selectivity and stereoselectivity toward the reduction of β-ketoesters. Tetrahedron..

[37] Zhang R, Xu Y, Xiao R, Zhang B, Wang L (2014). Optimized expression of (*S*)-carbonyl reductase in *Pichia pastoris* for efficient production of (*S*)-1-phenyl-1, 2-ethanediol. J Basic Microbiol.

[38] Yoshimoto M, Sato M, Yoshimoto N, Nakao K (2008). Liposomal encapsulation of yeast alcohol dehydrogenase with cofactor for stabilization of the enzyme structure and activity. Biotechnol Prog.

[39] Yoshimoto M (2011). Stabilization of enzymes through encapsulation in liposomes. Methods Mol Biol.

[40] Chandrawati R, Odermatt PD, Chong S-F, Price AD, Städler B, Caruso F (2011). Triggered cargo release by encapsulated enzymatic catalysis in capsosomes. Nano Lett.

[41] Nasseau M, Boublik Y, Meier W, Winterhalter M, Fournier D (2001). Substrate-permeable encapsulation of enzymes maintains effective activity, stabilizes against denaturation, and protects against proteolytic degradation. Biotechnol Bioeng..

[42] Urabe Y, Shiomi T, Itoh T, Kawai A, Tsunoda T, Mizukami F, Sakaguchi K (2007). Encapsulation of hemoglobin in mesoporous silica (FSM)-enhanced thermal stability and resistance to denaturants. ChemBioChem.

[43] Pandya PH, Jasra RV, Newalkar BL, Bhatt PN (2005). Studies on the activity and stability of immobilized α-amylase in ordered mesoporous silicas. Microporous Mesoporous Mater.

[44] Kloimwieder A, Winston F (2011). A screen for germination mutants in *Saccharomyces cerevisiae*. G3.

[45] Zhang RZ, Geng YW, Xu Y, Zhang WC, Wang SS, Xiao R (2011). Carbonyl reductase SCRII from *Candida parapsilosis* catalyzes anti-Prelog reaction to (*S*)-1-phenyl-1,2-ethanediol with absolute stereochemical selectivity. Bioresour Technol..

