# Supplementary material for: Efficient chiral synthesis by Saccharomyces cerevisiae spore encapsulation of Candida parapsilosis Glu228Ser/(S)-carbonyl reductase II and Bacillus sp. YX-1 glucose dehydrogenase in organic solvents
Source: Microb Cell Fact. 2019 May 20;18:87. doi: 10.1186/s12934-019-1137-6 (PMC6526602; doi:10.1186/s12934-019-1137-6)
Supplement: Supplementary file 1 — Additional file 1: Figure S1. Microscope images of enzyme-encapsulated spores cultured with YPD and reaction mixture. Figure S2. Optimal temperature and pH value on enantioselective synthesis of (R)-PE by SporeE228S-GDH. Table S1. Strains and plasmids in this study. Table S1. Strains, plasmids and primers in this study. Table S2. Analytical methods of corresponding chiral products. [file 12934_2019_1137_MOESM1_ESM.doc]

**Additional file 1**

**Efficient chiral synthesis by *Saccharomyces cerevisiae* spore encapsulation of *Candida parapsilosis* Glu228Ser/(*S*)-carbonyl reductase II and *Bacillus* sp. YX-1 glucose dehydrogenase in organic solvents**

Jingxin Rao^a^, Rongzhen Zhang^b^*, Hongbo Liang ^b^, Xiao-Dong Gao^c^, Hideki Nakanishi^c^, Yan Xu^b^

^a^ College of Science of China Pharmaceutical University, Nanjing 2111198, P. R. China

^b^ Key Laboratory of Industrial Biotechnology of Ministry of Education & School of Biotechnology, Jiangnan University, Wuxi 214122, P. R. China

^c^ Key Laboratory of Carbohydrate Chemistry and Biotechnology, Ministry of Education, School of Biotechnology, Jiangnan University, Wuxi, China

***Corresponding authors: Rongzhen Zhang,** [**rzzhang@jiangnan.edu.cn**](mailto:rzzhang@jiangnan.edu.cn)

**Tel: +86-510-85197760; Fax: +86-510-85864112**

**Present address: School of Biotechnology, Jiangnan University, 1800 Lihu Avenue, Wuxi 214122, P. R. China**

**Figure S1.** Microscope images of enzyme-encapsulated spores cultured with YPD and reaction mixture.

The spores quickly entered vegetative growth state and produced daughter-cells when they were cultured in YPD medium, while they maintained dormant state over 30 h in reaction mixture with the addition of 40 mM cycloheximide to the reaction mixture to prevent spore germination for each new reaction.


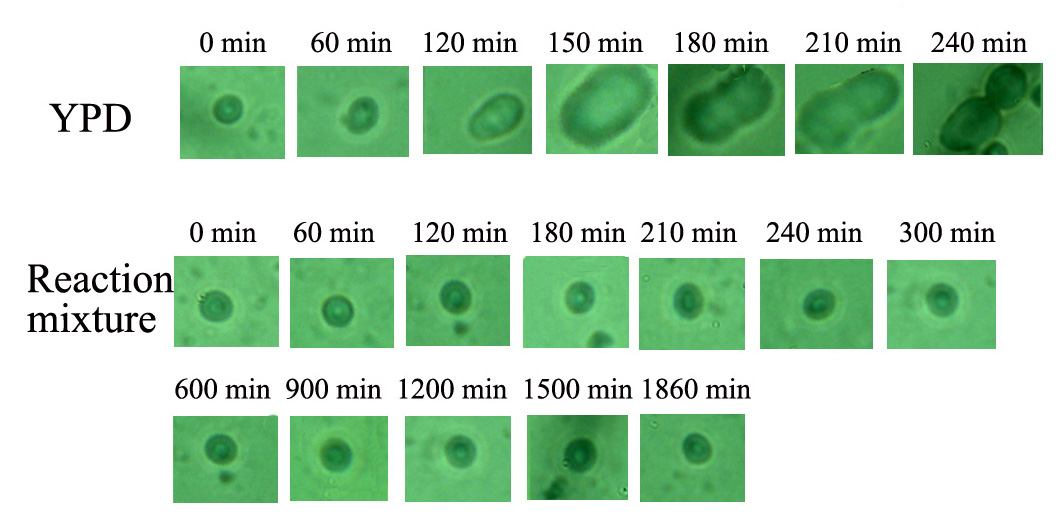


**Figure S2.** Observation of the green fluorescence distribution in *S. cerevisiae osw2*Δ/GFP-E228S-SD-AS-G. GFP-E228S-SD-AS-G expressed in *S. cerevisiae* AN120 *osw*2∆ cells were sporulated and intact asci were observed under the fluorescent or bright-field (BF) microscopy (×400). Images of *S. cerevisiae osw2*Δ spores with the plasmid pRS424-*TEF_pr_* are shown as a control (CK).

**
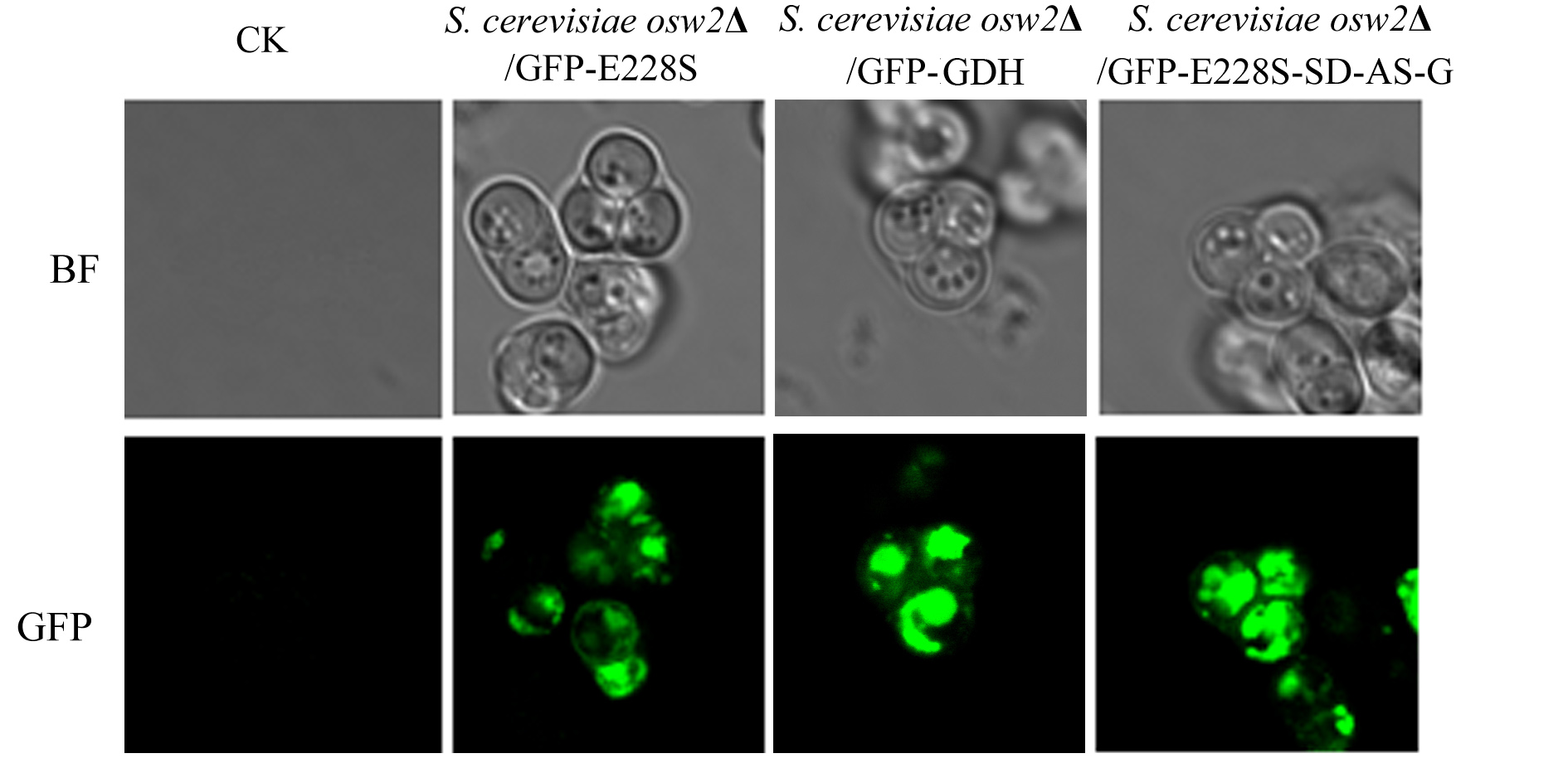
**

**Figure S3.** Optimal temperature and pH value on enantioselective synthesis of (*R*)-PE by Spore_E228S-GDH_.

**
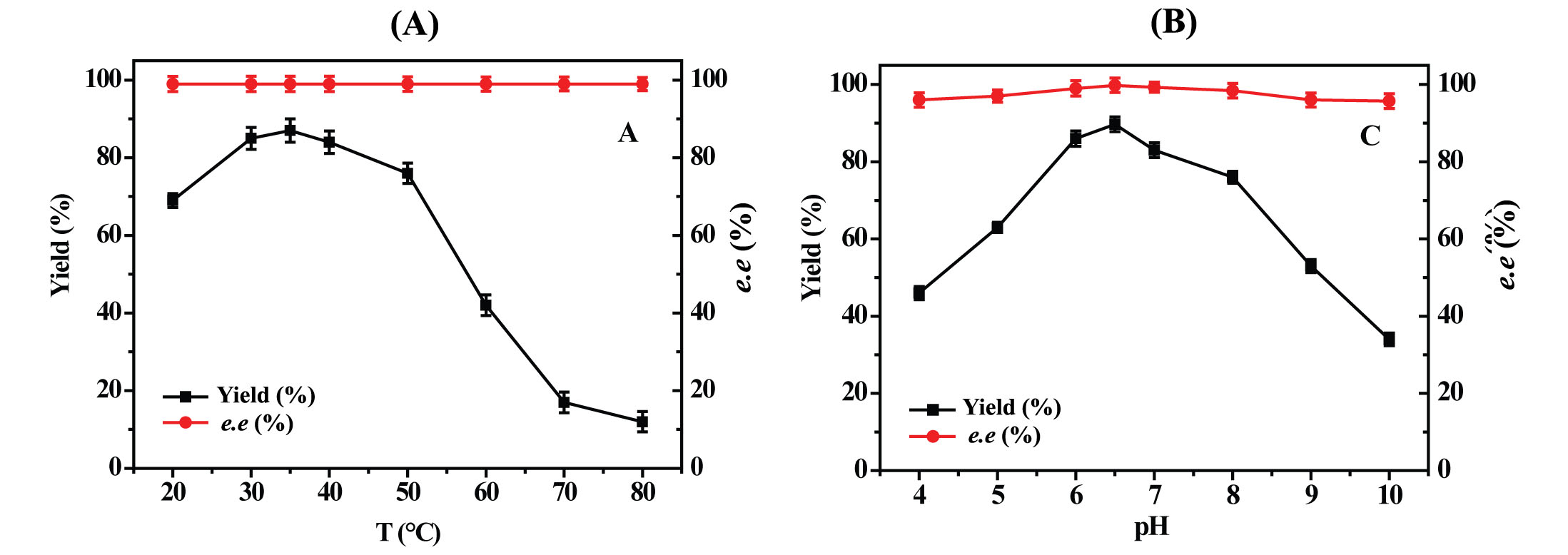
**

**Table S1** Strains, plasmids and primers in this study.

| Strains, plasmids and primers | | Description | Source |
| --- | --- | --- | --- |
| Strains | |  |  |
| *E*. *coli* JM109 | | r endA1 glnV44 thi-1 relA1 gyrA96 recA1 mcrB^+^ Δ(lac-proAB) e14- [F' traD36 proAB^+^ lacI^q^ lacZΔM15] hsdR17(r_K_^-^m_K_^+^) | Invitrogen |
| *E*. *coli* BL21 | | F^–^ ompT gal dcm lon hsdS_B_ (r_B_^–^m_B_^–^) [malB^+^]_K-12_(λ^S^) | Invitrogen |
| *S. cerevisiae* AN120 | | MATα/MATa ARG4/arg4-NspI his3∆SK/his3∆SK ho::LYS2/ho::LYS2 leu2/leu2 lys2/lys2 RME1/rme1::LEU2 trp1::hisG/trp1::hisG ura3/ura3 | This lab |
| *S. cerevisiae* AN120 *osw2*Δ | | MATα/MATa ARG4/arg4-NspI his3∆SK/his3∆SK ho::LYS2/ho::LYS2 leu2/leu2 lys2/lys2 RME1/rme1::LEU2 trp1::hisG/trp1::hisG ura3/ura3 osw2∆::his5+/osw2∆::his5+ | This lab |
| *E*. *coli* BL21/e228s | | Source of E228S | Zhang et al. 2013 |
| *E*. *coli* BL21/E228S-SD-AS-G | | Source of E228S-SD-AS-G |
| *E*. *coli* /E228S | | *E*. *coli* JM109 harboring pRS424-*TEF_pr_*-e228s, Amp^r^ | This study |
| *E*. *coli* /E228S-SD-AS-G | | *E*. *coli* JM109 harboring pRS424-*TEF_pr_*-e228s-SD-AS-G, Amp^r^ | This study |
| *E*. *coli* /GFP-E228S-SD-AS-G | | *E*. *coli* JM109 harboring pRS424-*TEF_pr_*-GFP-e228s-SD-AS-G, Amp^r^ | This study |
| *S. cerevisiae osw2*Δ/E228S | | *S. cerevisiae* AN120 harboring pRS424-*TEF_pr_*-e228s | This study |
| *S. cerevisiae osw2*Δ/GDH | | *S. cerevisiae* AN120 harboring pRS424-*TEF_pr_*-GDH | This study |
| *S. cerevisiae osw2*Δ/E228S-SD-AS-G | | *S. cerevisiae* AN120 *osw*2∆ harboring pRS424-*TEF_pr_*-e228s-SD-AS-G | This study |
| *S. cerevisiae osw2*Δ/GFP-E228S | | *S. cerevisiae* AN120 *osw*2∆ harboring pRS424-*TEF_pr_*-e228s | This study |
| *S. cerevisiae osw2*Δ/GFP-GDH | | *S. cerevisiae* AN120 *osw*2∆ harboring pRS424-*TEF_pr_*-GDH | This study |
| *S. cerevisiae osw2*Δ/GFP-E228S-SD-AS-G | | *S. cerevisiae* AN120 *osw*2∆ harboring pRS424-*TEF_pr_*-GFP-e228s-SD-AS-G | This study |
| Plasmids | |  |  |
| pRS424-*TEF_pr_* | | Expression vector of gene in *S. cerevisiae* AN120 | Shi et al. 2014 |
| pRS424-*TEF_pr_*-GFP | | Expression vector of gene in *S. cerevisiae* AN120 |
| pRS424-*TEF_pr_*-e228s | | pRS424-*TEF_pr_* harboring e228s | This study |
| pRS424-*TEF_pr_*-GDH | | pRS424-*TEF_pr_* harboring GDH | This study |
| pRS424-*TEF_pr_*-e228s-SD-AS-G | | pRS424-*TEF_pr_* harboring E228S-SD-AS-G | This study |
| pRS424-*TEF_pr_*-GFP-E228S | | pRS424-*TEF_pr_* -GFP harboring E228S | This study |
| pRS424-*TEF_pr_*-GFP-GDH | | pRS424-*TEF_pr_* -GFP harboring GDH | This study |
| pRS424-*TEF_pr_*-GFP-e228s-SD-AS-G | | pRS424-*TEF_pr_* -GFP harboring E228S-SD-AS-G | This study |
| Primers | 5’→ 3’ | |  |
| E228S_F | TGCACTGCAGATGGGCGAAATCGAATCTTATTGCA (*Pst* I) | | |
| E228S_R | CCGCTCGAGCTATGGACAAGTGTAACCACCAGC (*Xho* I) | | |
| Couple_F | TGCACTGCAGATGGGCGAAATCGAATCTTATTGCA (*Pst* I) | | |
| Couple_R | CCGCTCGAGTTAACCGCGGCCTGCCTGGAGAC (*Xho* I) | | |

Notes: The restriction endonuclease sites are underlined. Amp^r^ means ampicilin resistence.

**Table S2.** Analytical methods of corresponding chiral products.


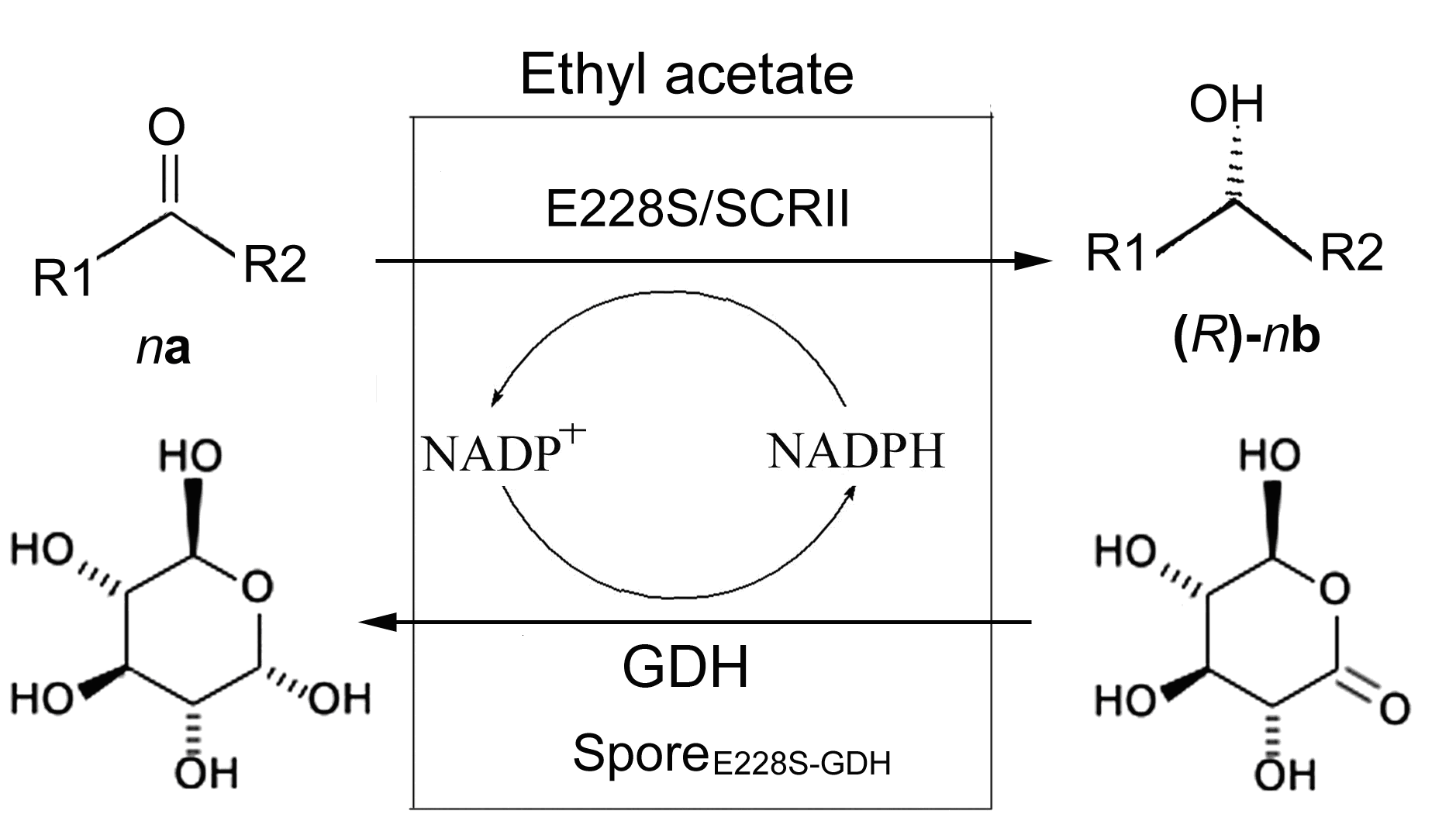


| Substrates | R1 | R2 | Methods | Mobile phase | | Column  Temperature(°C) | Flow rate/ mL·min^-1^ | | Peak time / min | |  | |
| --- | --- | --- | --- | --- | --- | --- | --- | --- | --- | --- | --- | --- |
|  |  |  |  |  |  |  |  |  | t_R_ | t_S_ |  | |
| 1a | Ph | CH_3_ | HPLC | Hexane:isopropanol 90:10 | | 25 | 0.6 | | 16.9 | 12.4 |  | |
| 2a | Ph-*p*-CH_3_ | CH_3_ | HPLC | Hexane:isopropanol 90:10 | | 25 | 0.4 | | 18.7 | 16.5 |  | |
| 3a | Ph-*p*-OCH_3_ | CH_3_ | HPLC | Hexane:isopropanol 90:10 | | 25 | 0.8 | | 21.1 | 24.2 |  | |
| 4a | Ph-*p*-Br | CH_3_ | HPLC | Hexane:isopropanol 98:2 | | 25 | 0.8 | | 21.1 | 24.2 |  | |
| 5a | Ph-*p*-Cl | CH_3_ | HPLC | Hexane:isopropanol 98:2 | | 25 | 0.8 | | 19.5 | 22.1 |  | |
| 6a | Ph-*o*-Cl | CH_3_ | HPLC | Hexane:isopropanol 98:2 | | 25 | 0.8 | | 13.3 | 19.8 |  | |
| 7a | Ph-*m*-Cl | CH_3_ | HPLC | Hexane:isopropanol 98:2 | | 25 | 0.8 | | 19.3 | 28.2 |  | |
| 8a | Ph | CH_2_OH | HPLC | Hexane:isopropanol 90:10 | | 38 | 0.4 | | 19.7 | 23.9 |  | |
| 9a | CH_3_(CH_2_)_2_ | CH_3_ | GC | Nitrogen | | 150°C for 7 min | - | | 4.96 | 5.07 |  | |
| 10a | CH_3_(CH_2_)_3_ | CH_3_ | GC | Nitrogen | | Oven program 60°C for 5 min then 15°C∙min^-1^ to 150°C for 7 min | - | | 15.41 | 15.16 |  | |
| 11a | CH_3_(CH_2_)_4_ | CH_3_ | GC | Nitrogen | | 160°C for 10 min | - | | 8.71 | 8.47 |  | |
| 12a | CH_3_(CH_2_)_5_ | CH_3_ | GC | Nitrogen | | Oven program 120°C for 2 min then 8°C∙min^-1^ to 200°C for 2 min | - | | 12.25 | 12.07 |  | |
| 13a | CH_3_COO | O-CH_3_ | GC | Nitrogen | | Oven program 60°C for 3 min then 2°C∙min^-1^ to 100°C, then 20°C∙min^-1^ to 200°C for 2 min | - | | 12.25 | 12.07 |  | |
| 14a | CCl_3_COCH_2_ | O-CH_2_CH_3_ | HPLC | Hexane:isopropanol 90:10 | | 25 | 0.5 | | 20.9 | 17.9 |  | |
| 15a | Ph-CO(CH_2_)_2_ | O-CH_3_ | HPLC | Hexane:isopropanol 80:20 | | 20 | 0.8 | | 5.9 | 7.3 |  | |
|  | | | | |  | | |  | | | | 1. *Alcohols were present in Figure. 2-2* |

**References:**

Shi, L., Li, Z., Tachikawa, H., Gao, X., Nakanishi, H., 2014. Microencapsulation of enzymes using yeast spores. Appl. Environ. Microbiol., 80, 4502-4510.

Zhang, R., Zhang, B., Xu, Y., Li, Y., Li, M., Liang, H., Xiao, R., 2013. Efficicent (R)-phenylethanol production with enantioselectivity-alerted (S)-carbonyl reductase II and NADPH regeneration. Plos One. 8, e83586.
